# A comparative analysis reveals weak relationships between ecological factors and beta diversity of stream insect metacommunities at two spatial levels

**DOI:** 10.1002/ece3.1439

**Published:** 2015-02-23

**Authors:** Jani Heino, Adriano S Melo, Luis Mauricio Bini, Florian Altermatt, Salman A Al-Shami, David G Angeler, Núria Bonada, Cecilia Brand, Marcos Callisto, Karl Cottenie, Olivier Dangles, David Dudgeon, Andrea Encalada, Emma Göthe, Mira Grönroos, Neusa Hamada, Dean Jacobsen, Victor L Landeiro, Raphael Ligeiro, Renato T Martins, María Laura Miserendino, Che Salmah Md Rawi, Marciel E Rodrigues, Fabio de Oliveira Roque, Leonard Sandin, Denes Schmera, Luciano F Sgarbi, John P Simaika, Tadeu Siqueira, Ross M Thompson, Colin R Townsend

**Affiliations:** 1 Finnish Environment Institute, Natural Environment Centre, BiodiversityOulu, Finland; 2 Departamento de Ecologia, Universidade Federal de GoiásGoiânia, GO, Brazil; 3 Department of Aquatic Ecology, Eawag: Swiss Federal Institute of Aquatic Science and TechnologyDübendorf, Switzerland; 4 Institute of Evolutionary Biology and Environmental Studies, University of ZurichZürich, Switzerland; 5 School of Biological Sciences, Universiti Sains MalaysiaPenang, Malaysia; 6 Biology Department, Faculty of Science, University of TabukTabuk, Saudi Arabia; 7 Department of Aquatic Sciences and Assessment, Swedish University of Agricultural SciencesUppsala, Sweden; 8 Departament d'Ecologia, Grup de Recerca Freshwater Ecology and Management (FEM), Universitat de BarcelonaBarcelona, Catalonia, Spain; 9 LIESA-CONICET-Universidad Nacional de la Patagonia SJBChubut, Argentina; 10 Departamento de Biologia Geral, Instituto de Biologia Geral, Universidade Federal de Minas GeraisBelo Horizonte, Minas Gerais, Brazil; 11 Department of Integrative Biology, University of GuelphGuelph, ON, Canada; 12 Laboratory of Entomology, School of Biological Sciences, Pontifical Catholic University of EcuadorQuito, Ecuador; 13 IRD, Institut de Recherche pour le Développement, Laboratoire Evolution, Génomes et SpéciationGif-sur-Yvette, France; 14 School of Biological Sciences, The University of Hong KongHong Kong SAR, China; 15 Laboratorio de Ecología Acuática Colegio de Ciencias Biológicas y Ambientales Universidad San Francisco de QuitoQuito, Ecuador; 16 Department of Bioscience, Aarhus UniversitySilkeborg, Denmark; 17 Instituto Nacional de Pesquisas da Amazônia, Coordenação de BiodiversidadeManaus, AM, Brazil; 18 Department of Biology, University of CopenhagenCopenhagen, Denmark; 19 Departamento de Botânica e Ecologia, Universidade Federal do Mato GrossoCuiabá, Brazil; 20 Departamento de Ciências Biológicas e da Saúde, Universidade Federal de Mato Grosso do SulCampo Grande, Mato Grosso do Sul, Brazil; 21 Section of Conservation Biology, Department of Environmental Sciences, University of BaselBasel, Switzerland; 22 Balaton Limnological Institute, Centre for Ecological Research, Hungarian Academy of SciencesTihany, Hungary; 23 Department of Conservation Ecology and Entomology, Stellenbosch UniversityStellenbosch, South Africa; 24 Instituto de Biociências, UNESP - Universidade Estadual PaulistaRio Claro, São Paulo, Brazil; 25 Institute for Applied Ecology, University of CanberraCanberra, ACT, Australia; 26 Department of Zoology, University of OtagoDunedin, New Zealand

**Keywords:** Altitude range, comparative analysis, environmental filtering, insects, latitude, spatial extent, variance partitioning

## Abstract

The hypotheses that beta diversity should increase with decreasing latitude and increase with spatial extent of a region have rarely been tested based on a comparative analysis of multiple datasets, and no such study has focused on stream insects. We first assessed how well variability in beta diversity of stream insect metacommunities is predicted by insect group, latitude, spatial extent, altitudinal range, and dataset properties *across multiple drainage basins* throughout the world. Second, we assessed the relative roles of environmental and spatial factors in driving variation in assemblage composition *within each drainage basin*. Our analyses were based on a dataset of 95 stream insect metacommunities from 31 drainage basins distributed around the world. We used dissimilarity-based indices to quantify beta diversity for each metacommunity and, subsequently, regressed beta diversity on insect group, latitude, spatial extent, altitudinal range, and dataset properties (e.g., number of sites and percentage of presences). Within each metacommunity, we used a combination of spatial eigenfunction analyses and partial redundancy analysis to partition variation in assemblage structure into environmental, shared, spatial, and unexplained fractions. We found that dataset properties were more important predictors of beta diversity than ecological and geographical factors across multiple drainage basins. In the within-basin analyses, environmental and spatial variables were generally poor predictors of variation in assemblage composition. Our results revealed deviation from general biodiversity patterns because beta diversity did not show the expected decreasing trend with latitude. Our results also call for reconsideration of just how predictable stream assemblages are along ecological gradients, with implications for environmental assessment and conservation decisions. Our findings may also be applicable to other dynamic systems where predictability is low.

## Introduction

The importance of understanding broad-scale patterns of biodiversity is becoming ever more urgent as landscapes, ecosystems, and communities are increasingly transformed by processes such as habitat loss, invasion by exotic species, eutrophication, and climate change (Dudgeon et al. 2006; Vörösmarty et al. 2010). Increased understanding of broad-scale biodiversity patterns is a prerequisite for the advancement of both basic and applied ecology. From the perspective of basic ecology and for the sake of generality, more information on biodiversity patterns of a variety of under-studied taxa is needed (Leather 2009). From the perspective of applied ecology, knowledge of patterns in biodiversity is essential to guide both conservation actions and environmental assessment (Caro 2010). Accordingly, better understanding of patterns in regional species richness (*γ*-diversity), local species richness (*α*-diversity), and variation in species composition between sites (*β*-diversity) at the global, continental, and regional scales would be valuable (Heino 2013; Bini et al. 2014).

Hypotheses about the processes driving beta diversity are closely intertwined with recent developments in metacommunity theory (Leibold et al. 2004; Logue et al. 2011). While variation in local community composition is thought to be typically driven by species sorting along environmental gradients (Cottenie 2005), spatial processes (e.g., dispersal between sites) also have the potential to affect local community composition (Brown and Swan 2010; Altermatt et al. 2013; Heino and Peckarsky 2014; Heino et al. 2015a). The relative importance of species sorting versus spatial processes may be contingent on the lengths of environmental gradients (e.g., range in stream temperature or nutrient concentrations within a drainage basin) and the spatial extent of the study (Jackson et al. 2001; Cottenie 2005; Bini et al. 2014; Heino et al. 2015a). One might expect species sorting to increase with increasing environmental gradient length (Jackson et al. 2001; Grönroos et al. 2013) and spatial factors to gain importance with increasing spatial extent of the region studied (Cottenie 2005; Heino 2011). Very few studies have explicitly tested these hypotheses using multiple datasets, which is surprising given that environmental heterogeneity and spatial scale are two key ideas behind metacommunity theory (Leibold et al. 2004). While the importance of species sorting and spatial factors for metacommunity organization can be addressed in single case studies, such studies do not allow the identification of robust patterns and cannot lead to broad generalizations. Instead, the relative influence of environmental heterogeneity (cf. species sorting) and spatial extent (cf. spatial factors) can only be investigated in a general sense across multiple metacommunities in different geographic regions using a comparative approach. Such broad-scale analyses have seldom involved stream organisms, and the analyses that have been undertaken have either focused on a small component of benthic insect assemblages (Boyero et al. 2012) or on specific stream types (Jacobsen and Dangles 2012).

Running waters offer an ideal model system to disentangle the relative influences of species sorting and spatial processes on metacommunity organization (Brown et al. 2011; Heino et al. 2015b). Stream ecosystems show high geomorphological heterogeneity (Allan and Castillo 2007), are structured in dendritic networks (Altermatt 2013), and harbor exceptional levels of biodiversity relative to their limited spatial extent (Dudgeon et al. 2006). Aquatic insects are prominent organisms in streams, playing key roles in food webs and ecosystem processes (Allan and Castillo 2007) and showing high diversity in terms of phylogenetic origins, dispersal traits, species richness, and endemism (Lancaster and Downes 2013). However, they are assumed to be strongly impacted by various anthropogenic factors, including pollution (Rosenberg and Resh 1993), habitat modification (Allan and Castillo 2007), and climate change (Jacobsen et al. 2012). This sensitivity potentially makes stream insects valuable as biological indicators (Rosenberg and Resh 1993) and, hence, understanding patterns and scales of variability in their metacommunities is a priority (Heino 2013).

Current knowledge on stream insect metacommunities mainly relies on case studies conducted in individual regions (e.g., Thompson and Townsend 2006; Heino and Mykrä 2008; Brown and Swan 2010; Landeiro et al. 2012; Siqueira et al. 2012; Al-Shami et al. 2013a). Most studies have shown that species sorting is prevalent in shaping these metacommunities, although the strength of dispersal limitation may increase with increasing spatial scale (Heino and Peckarsky 2014). The mechanisms structuring metacommunities may also be contingent on system-specific factors (e.g., high dispersal rates may be important in mainstem rivers; Brown and Swan 2010) or be related to dispersal traits (e.g., the importance of dispersal limitation increases with decreasing dispersal ability; Thompson and Townsend 2006; Heino et al. 2015b). These and other hypotheses associated with broad-scale patterns are difficult to test with a single or a few datasets, and more general perspectives may be obtained through a comparative analysis of multiple datasets. Using a large number of stream insect datasets from different parts of the world, we attempted to reveal the main factors structuring stream insect metacommunities by answering the following questions: (Q1) How well can variability in beta diversity *across* multiple stream insect metacommunities (i.e., spatial level 1; Appendix S1) be accounted for by insect group, latitude, altitudinal range, and spatial extent? (Q2) What are the relative roles of species sorting and spatial processes in predicting assemblage composition *within* stream insect metacommunities (i.e., spatial level 2; Appendix S2)?

We assembled a comprehensive dataset comprising 95 stream insect metacommunities from different regions, ranging from tropical to Arctic latitudes and lowland to montane habitats. First, we analyzed spatial variation in beta diversity, with the expectation of higher beta diversity in tropical regions than nearer the poles (Soininen et al. 2007; but see Boyero et al. 2011). Second, we tested whether the importance of environmental factors in accounting for variation in assemblage composition tended to be greater within drainage basins that had higher environmental variability (Heino 2011; Grönroos et al. 2013), and whether spatial factors increased in importance with increasing regional extent (Cottenie 2005; Bini et al. 2014). We showed that beta diversity patterns may be poorly predictable in highly dynamic stream systems, which calls for reconsideration of the predictability of ecological communities.

## Methods

### Study areas and datasets

**Figure 1:**
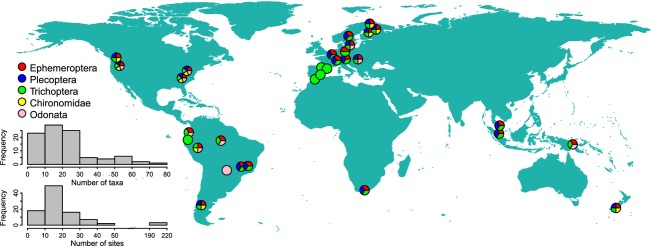
Geographical locations of the 31 drainage basins in this study. Analyses were carried out for each insect taxon separately. Thereby, 95 datasets were available for analyses of biological data only and 61 datasets for analyses using environmental predictors. In some cases, symbols have been shifted slightly to avoid overlap. The inset histograms show the frequency distribution of number of species (upper histogram) and number of sites per metacommunity (lower histogram).

We assembled data for five major stream insect taxa: mayflies (Ephemeroptera), stoneflies (Plecoptera), caddisflies (Trichoptera), nonbiting midges (Diptera: Chironomidae), and dragonflies (Odonata) from 31 drainage basins distributed around the world, but predominantly from the Americas and Europe (Fig.1). These five insect groups often dominate in stream invertebrate communities (Lancaster and Downes 2013; Heino and Peckarsky 2014), and the family Chironomidae may alone account for a very large share of species in a dataset (e.g., Raunio et al. 2011). However, given the lack of data for other important taxa, such as beetles (Coleoptera), our results are only applicable to the five taxonomic groups analyzed. Both insect and environmental data were available for 23 basins, while insect data only were available for the remaining eight basins. We initially intended to use datasets consisting of at least 15 stream sites per basin, but included a few basins with a lower number of sites to increase geographic coverage for across-basins beta diversity analyses (see below). The average number of sites per drainage basin was 24.5 (range: 4 to 213), and most drainage basins included data for at least three insect taxonomic groups (see below) (Fig.1). Each insect dataset was also checked to ensure that surveys were conducted within a single year and that sampling did not span multiple seasons (i.e., samples within a drainage basin were taken within a short period of time). This was performed to guarantee that we were studying sets of potentially interacting species (see Leibold et al. 2004). In addition, each insect dataset was based on standardized sampling methods (e.g., kick-netting or Surber samplers), and between-site differences in species composition were thus comparable within each dataset. We focused on datasets from streams subject to little anthropogenic impact, although in a few basins, some streams were affected by adjacent human land use (e.g., some streams drained managed forests).

Insect data for each drainage basin were separated by taxonomic group for analyses, yielding a total of 95 datasets, of which 75 included both insect and environmental data. However, in the within-basin analyses described below, we used only 61 datasets that included at least 15 sites. We used this approach because each taxonomic group may show distinct latitudinal patterns in diversity (Vinson and Hawkins 2003; Pearson and Boyero 2009), exhibit different dispersal abilities (Bonada et al. 2012), or respond differently to environmental factors (Heino and Mykrä 2008). Furthermore, different taxonomic groups may exhibit different patterns of metacommunity organization (Heino and Mykrä 2008; Altermatt et al. 2013).

Taxonomic resolution varied among the insect datasets due to differences in taxonomic knowledge among regions; however, all datasets involved identification to at least the level of genus. We also controlled for regional differences in taxonomic knowledge by including the variable “proportion of taxa identified to species level” in analyses comparing variability in beta diversity *across* metacommunities. We had to rely on incidence (i.e., presence–absence) data because we did not have strictly comparable abundance data from all drainage basins. Although numerical resolution may affect patterns in beta diversity (Anderson et al. 2011; Heino et al. 2015a), previous studies on stream invertebrates have shown that the main patterns can be reproduced using either presence–absence or abundance data (Al-Shami et al. 2013b; Heino et al. 2013; Bini et al. 2014).

### Environmental variables

The physical and chemical variables that were measured in each drainage basin varied greatly (Appendix S3). We removed some categorical variables from drainage basin datasets where there were fewer sites than explanatory variables. We also pooled fine and coarse estimates of benthic organic matter when both were present in the same dataset. Similarly, for datasets that included proportional coverage of substrate size classes, the finer categories (i.e., silt, sand, gravel, and pebble) were pooled. We also removed variables that provide similar information (e.g., we retained only total nitrogen when several forms of nitrogen were reported), as well as variables only measured at a few sites within a drainage basin and some variables related to riparian vegetation (e.g., width of the riparian vegetation) that affect stream insects indirectly.

After this initial data screening, we finalized environmental datasets for each insect group in each drainage basin. This involved deletion of sites from which a particular insect group was absent and deletion of variables for which observations were only available for a few sites. Finally, we removed entire datasets including <15 sites. The 61 final environmental datasets were derived from 20 basins and included 4–21 variables, and a total of 67 variables were used. Conductivity, total phosphorus, and depth were the most common variables, available for 54, 41, and 40 datasets, respectively (Appendix S3).

### Beta diversity analyses

Multiple approaches are necessary to adequately describe patterns in beta diversity, because each approach may provide distinct information about this facet of biodiversity (Anderson et al. 2011). We used three approaches to estimate beta diversity for each of the 95 metacommunities. The first was the average of pairwise dissimilarities between sites within a drainage basin. The second was the multiple-site version of the same metric (Diserud and Ødegaard 2007), which is a generalization of the 2-samples formula to handle more than two samples. The third was the average biological distance of sites within a single metacommunity to the metacommunity centroid (Anderson et al. 2006). Each of these three approaches was based on three different dissimilarity coefficients: (i) Sørensen coefficient (i.e., a measure of overall beta diversity), (ii) Simpson coefficient (i.e., a measure of turnover immune to nestedness resulting from species richness differences), and (iii) a coefficient measuring nestedness resulting from species richness differences (Baselga 2010).

### Comparative analyses of beta diversity

**Figure 2:**
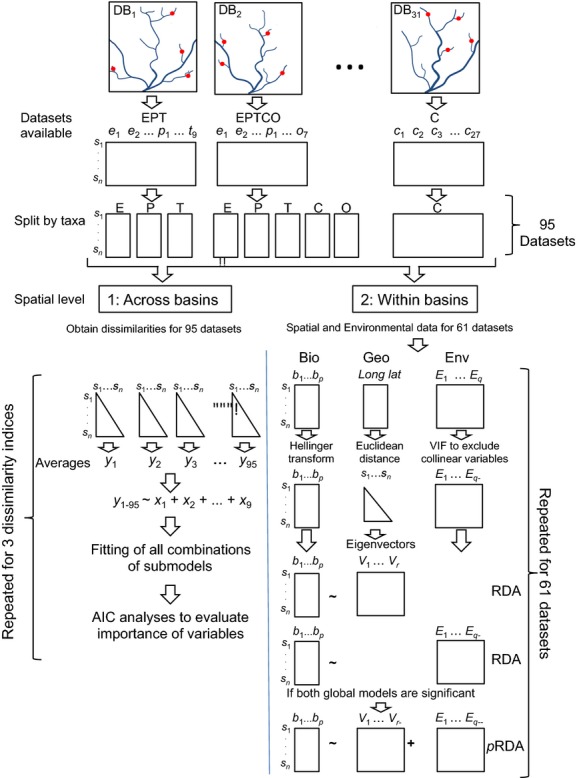
Flow chart of the statistical analyses used. Different analyses were employed at (1) the across-basins level and (2) the within-basin level. See main manuscript text for details. DB, Drainage basin; E, Ephemeroptera; P, Plecoptera; T, Trichoptera; C, Chironomidae; O, Odonata; Bio, biological data, Env, environmental data; Geo, geographic coordinates. *s*, sites; *n*, number of sites; y, averages of pairwise dissimilarities (from the first to the 95th dataset); x_1-9_, explanatory variables for the across-basins analysis; *p*, number of taxa in a given biological data matrix; *q*, *q*_-_, and *q*_–_, number of environmental variables (*E*) before, after using VIF and after using forward selection, respectively; *r* and *r*_-_, number of eigenvectors variables (*V*) before and after using forward selection.

We regressed dissimilarity-based beta diversities obtained for each drainage basin (*n *=* *95) against (i) taxonomic group (as a dummy variable), (ii) latitude (absolute values), (iii) range in altitude, and (iv) spatial extent of a drainage basin. We also included (v) an interaction term between range in altitude and latitude (i.e., the product of range in altitude and latitude) because the effects of range in altitude may depend on latitude (Hawkins and Diniz-Filho 2006). In addition to these factors, estimates of beta diversity may be influenced by the properties of the datasets (Podani and Schmera 2011). Thus, we also included in the regression models the following predictor variables: (vi) number of sites in the dataset, (vii) number of species, (viii) matrix fill (percentage of presences), and (ix) percentage of taxa identified to species level. Three datasets included many more stream sites than others (Fig.1), so we log-transformed this variable to improve the distribution of residuals. See Figure2 for the main steps of our statistical approach.

We hypothesized that beta diversity should be associated with environmental heterogeneity (Anderson et al. 2006; Heino et al. 2013). However, a variable directly related to environmental heterogeneity could not be estimated for all datasets because only biological data were available for some of them. Also, the number and type of environmental variables measured in each basin varied. We therefore assessed whether range in altitude could serve as a proxy for environmental variation among stream sites within a drainage basin. We obtained altitudinal range for each dataset from the Shuttle Radar Topography Mission (Jarvis et al. 2008). We then calculated the average coefficient of variation for stream environmental variables within each dataset and correlated it with altitudinal range, revealing a positive correlation (*r *=* *0.55). We thus opted to use altitudinal range as a surrogate variable for environmental variability or heterogeneity because this metric allowed us to include all 95 datasets in the comparative analyses rather than only the 75 (i.e., also including datasets with <15 sites) for which environmental data were available. Altitudinal range is likely to be related to variation in multiple environmental factors affecting the distribution of stream organisms, including temperature, oxygen concentration, and differences in habitat conditions between lowland and mountain streams (Ward 1998; Jacobsen and Dangles 2012). Hence, the larger the altitudinal range in a drainage basin, the larger the variation in stream environmental conditions organisms have to cope with.

Two metrics of spatial extent were obtained initially. The first was the area of a minimum convex polygon including all sites in the drainage basin. Although straightforward, this metric is expected to be biased in cases where a few sites are located far from most of the others. Accordingly, we also obtained the average distance of sites to the geographic centroid of all sites within a drainage basin. These two metrics were strongly correlated (*r *=* *0.84), and we opted to use the average distance of sites to the geographic centroid.

### Model evaluations

We calculated the second-order Akaike's information criterion (AIC) to rank the best approximating models explaining variation in our measures of beta diversity (Fig.2). AIC differences (AIC_*i*_ −AIC_*min*_, where AIC_*min*_ is the AIC for the best model in the set, given the data) were also calculated. AIC differences were then used to estimate the Akaike weight of each model (*w*_*i*_), which “may be interpreted as the probability that model *i* is the actual expected Kullback–Leibler distance best model for the sampling situation considered” (Burnham and Anderson 2002). The sum of Akaike weights over all models that included an explanatory variable (*w*_+_(*j*)) was calculated to estimate the relative importance of explanatory variables. We present the results for all models with AIC differences <2.0.

### Explaining variation in the biological datasets within each drainage basin

In each drainage basin, spatial variables were generated from the Euclidean distance matrix through Moran eigenvector maps (MEM), formerly called principal coordinates of neighbor matrices (Borcard and Legendre 2002). The spatial variables (eigenvectors) derived from MEM represent orthogonal patterns of relationships among sampling sites and are used as spatial predictor variables (Fig.2). Eigenvectors with high eigenvalues represent broad-scale patterns, whereas those associated with low eigenvalues represent fine-scale patterns. MEM thus produces multiple spatial variables that are efficient in capturing complex spatial patterns in the response data. As watercourse distances between sites were available in only a few datasets, we had to rely on using overland distances in MEM. This is likely to be appropriate because all of our taxa have flying adult stages. Although some studies have suggested that watercourse distances are more meaningful for stream organisms than overland distances (Altermatt et al. 2013), others have found that these two measures of distance are often strongly correlated, providing virtually the same information about the spatial structuring of a metacommunity (Thompson and Townsend 2006; Landeiro et al. 2012; Grönroos et al. 2013).

We used redundancy analysis (RDA; Legendre & Legendre, 2012) to examine the relative contributions of environmental and spatial variables (from MEM) to variation in assemblage composition (using abundance data when available) among sites. Partial RDA (pRDA) was employed to estimate fractions of the total variation of site-by-taxon matrices explained by environmental and spatial variables. The insect matrices were subjected to the Hellinger transformation that is suitable for both presence–absence and abundance data (Legendre et al. 2005), making assemblage data analyzable by linear ordination methods (Peres-Neto et al. 2006). We excluded environmental variables with variance inflation factors higher than 10 (Kutner et al. 2004). Then, we ran two global RDA models, one using all spatial predictors (i.e., eigenvectors) and the second using all remaining environmental predictors. If no global model was significant, the analysis was terminated, and the environmental and spatial fractions were assumed to be zero. When there was a significant global model, we ran a forward selection procedure to retain only the most important variables (Blanchet et al. 2008). In the forward selection, each variable retained should be significant at an alpha level of 0.05 and the adjusted *R*^2^ of the final RDA model should not be greater than its respective global model. When both spatial and environmental global models were significant, we ran a pRDA using the selected spatial and environmental variables to evaluate how much of the total biological variance each set of explanatory variables could explain. The variance partitioning relative to this pRDA was based on the adjusted *R*^*2*^ (Peres-Neto et al. 2006).

All analyses were undertaken in R (The R Core Team 2012). Dissimilarities and RDAs were obtained using functions from the *vegan* (Oksanen et al. 2012) and *betapart* (Baselga et al. 2013) packages. Candidate models were compared and model averaging performed using functions of the *MuMIn* package (Bartoń, 2014).

## Results

### Explaining dissimilarity-based beta diversity across drainage basins

Based on the Sørensen coefficient, beta diversity values showed high variation across the 95 datasets, ranging from 0.06 to 0.94 for the average of pairwise dissimilarities and from 0.14 to 0.99 for its multiple-sites version. Beta diversity quantified as the average of pairwise dissimilarities was strongly correlated to the average of distances to the metacommunity centroid (*r *=* *0.99). The average of pairwise dissimilarities was also correlated to the multiple-sites version, although to a lesser degree (*r *=* *0.75).

The correlation structure among the three beta diversity metrics based on the Simpson coefficient and among the three nestedness-resultant beta diversity metrics was similar to that found for the Sørensen coefficient. The average of pairwise dissimilarities of the Simpson coefficient was strongly correlated with the average of distances to the metacommunity centroid (*r *=* *0.95) and to a lesser extent to the multiple-sites version (*r *=* *0.76). For the nestedness-resultant beta diversity metrics, the average of pairwise dissimilarities was strongly correlated with the average of distances to the metacommunity centroid (*r *=* *0.89) and to a lesser extent with the multiple-sites version (*r *=* *0.67).

As all three dissimilarity-based beta diversity approaches were strongly correlated, regression results are shown for the average of pairwise dissimilarities only. The best models for the Sørensen-based beta diversity did not provide support for the hypothesized relationships with altitudinal range, insect group, or latitude (Appendix S4). The best models explained around 68% (adjusted *R*^2^) of the variance and included variables related to matrix properties, namely matrix fill, number of sites, and number of taxa (Tables1 and 2). In decreasing order, the six most important variables were matrix fill, number of sites, number of taxa, altitudinal range, latitude, and spatial extent (Table2). Similar results were found for the Simpson-based beta diversity, except for the inclusion of altitudinal range and proportion of taxa identified to species in some of the best models (Tables1 and 2). Regression results for nestedness-resultant beta diversity differed from those for Sørensen and Simpson indices (Tables1 and 2). A smaller set of models showed AIC_*c*_ differences >2.0, and they usually tended to fit the data poorly (adjusted *R*^2^ < 0.18).

**Table 1:** Summary of best models to explain variation in beta diversity quantified as average pairwise dissimilarities for each metacommunity. Models were obtained for Sørensen dissimilarity (total beta diversity), Simpson dissimilarity (beta diversity due to turnover), and nestedness dissimilarity resulting from richness differences. Full models included ecological variables hypothesized to have effects on dissimilarities, including (i) taxonomic group (group), (ii) latitude (absolute values), (iii) range in altitude (alt.rng), and (iv) spatial extent (spt.ext). We included (v) an interaction term because effects of range in altitude may depend on latitude. In addition to ecological factors, we included covariates related to matrix properties (vi) number of sites (log-transformed) (n.sites), (vii) number of species (n.spp), (viii) matrix fill (percentage of presences; fill), and (ix) percentage of taxa identified to species. Best models were selected according to the AICc statistics

	AICc	df	Delta	Weight	Adj. R^2^
Sørensen					
f'ill+n.sites	−178.8	4	0.00	0.373	0.684
fill+n.sites+n.spp	−177.8	5	1.01	0.225	0.685
fill+n.sites+spt.ext	−176.9	5	1.93	0.142	0.682
Simpson					
fill+n.sites+n.spp	−138.1	5	0.00	0.430	0.635
fill+n.sites+n.spp+prop.sp	−136.9	6	1.24	0.231	0.635
fill+n.sites+n.spp+alt.rng	−136.5	6	1.62	0.191	0.634
Richness-resultant					
n.sites+n.spp+prop.sp	−258.7	5	0.00	0.110	0.159
n.sites+n.spp	−258.7	4	0.04	0.108	0.148
n.sites+n.spp+alt.rng+spt.ext	−258.5	6	0.26	0.097	0.168
n.sites+n.spp+alt.rng+spt.ext+prop.sp	−258.1	7	0.63	0.081	0.176
n.sites+n.spp+spt.ext+prop.sp	−258.0	6	0.71	0.077	0.164
n.sites+n.spp+spt.ext	−257.7	5	1.05	0.065	0.150
n.sites+n.spp+alt.rng	−257.6	5	1.16	0.062	0.149
n.sites+n.spp+alt.rng+spt.ext+lat	−257.6	7	1.20	0.061	0.159
n.sites+n.spp+prop.sp+fill	−257.3	6	1.40	0.055	0.158
n.sites+n.spp+fill	−257.3	5	1.43	0.054	0.147
n.sites+n.spp+alt.rng+spt.ext+fill	−257.2	7	1.55	0.051	0.168
n.sites+n.spp+alt.rng+prop.sp	−257.1	6	1.60	0.050	0.156
n.sites+n.spp+spt.ext+prop.sp+fill	−257.0	7	1.70	0.047	0.166
n.sites+n.spp+alt.rng+spt.ext+prop.sp+fill	−256.9	8	1.88	0.043	0.176

df, degrees of freedom; Delta, AIC difference regarding the best model; Weight, Akaike weight; adj *R*^2^, ordinary adjusted coefficient of determination.

**Table 2:** Relative importance of predictor variables for pairwise Sørensen, Simpson and richness-resultant dissimilarities and standardized (beta) coefficients obtained from model averaging over all combinations of model terms. The insect taxon was a categorical variable with five levels coded as a dummy variable. The coefficient for Chironomidae was set to zero

	Sørensen	Simpson	Richness-resultant
Matrix fill	1.00	1.00	0.32
Relative importance of predictor variables			
Number of sites	0.99	0.99	0.99
Number of taxa	0.38	0.93	0.99
Altitudinal range	0.27	0.36	0.49
Latitude	0.26	0.32	0.34
Spatial extent	0.26	0.27	0.52
Proportion identified species	0.24	0.33	0.47
Insect group	0.05	0.17	0.11
Altitudinal range × Latitude	0.02	0.07	0.07
Model averaging			
Matrix fill	−0.927	−0.826	0.112
Number of sites	−0.268	−0.390	0.416
Number of taxa	0.081	0.219	−0.430
Altitudinal range	0.017	0.029	−0.131
Latitude	0.007	0.018	−0.042
Spatial extent	0.028	−0.039	0.175
Proportion identified species	0.008	0.068	−0.154
Insect taxon: Ephemeroptera	0.078	0.122	−0.050
Odonata	0.144	0.233	−0.229
Plecoptera	0.041	0.127	−0.149
Trichoptera	0.062	0.162	−0.223
Altitudinal range × Latitude	0.095	0.199	−0.234

Regardless of the measure of beta diversity used, the most important explanatory variables were more strongly related to matrix dimensions (number of sites and number of species) and dataset characteristics (especially matrix fill) than to biological, ecological, or geographical factors (Table2). There was also substantial uncertainty regarding the best models, as indicated by their Akaike weights and the evidence ratio between the best models (Table1). However, the weighted average of the estimates based on model uncertainty also suggested a greater importance of data properties as compared to the other explanatory variables (Table2).

### Explaining metacommunity variation within drainage basins: variance partitioning

RDAs indicated that about half of the insect datasets were associated with environmental or spatial predictors. Global RDA models for environmental predictors were significant (*P* < 0.05) for 28 of the 61 metacommunities. For spatial predictors, global RDA models were significant for 13 metacommunities.

**Figure 3:**
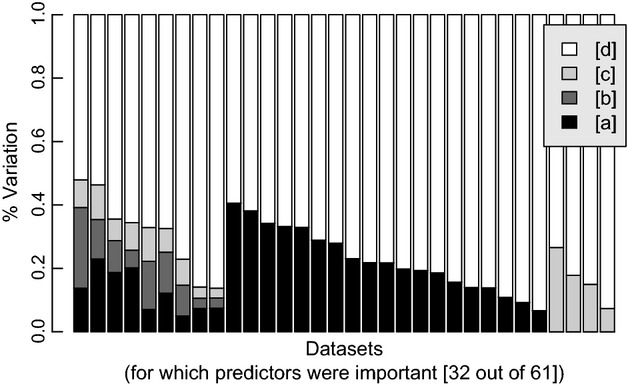
Variation partitioning of the 32 metacommunities for which environmental or spatial predictors were important. In the remaining 29 metacommunities, neither environmental nor spatial predictors significantly explained observed variation. Fractions [a], [b], [c], and [d] correspond to those due to environment, shared environment-space, space, and unexplained variation, respectively. Environmental and spatial predictors were both important for nine metacommunities and all four fractions obtained from partial redundancy analysis (pRDA) models are presented. Environmental or spatial predictors were important for 19 and 4 metacommunities, respectively, and their explained fractions obtained from RDA models. In all cases, a forward selection procedure was used to select predictor variables.

In only nine cases was metacommunity structure related to both environmental and spatial predictors (Fig.3). Among these, pRDA models obtained by forward selection indicated that the percentages of variation explained by the environmental variables (fraction [a]) and spatial variables (fraction [c]) were on average (mean ± SD) 13.0 ± 6.8% and 6.1 ± 3.1%, respectively. The average amount of variance shared by environmental and spatial variables [b] was 10.6 ± 6.2%. Total explained variance ([a] + [b] + [c]) by the forward-selected pRDA models was 29.7 ± 11.5% (Fig.3).

The structures of 19 metacommunities (28–9 = 19) were associated exclusively with environmental predictors, and four datasets (13–9 = 4) exclusively with spatial predictors (Fig.3). Among the former, environment explained on average 22.6 ± 10.0%. For the four datasets exclusively related to spatial predictors, variation explained was 16.7 ± 7.9%.

After exclusion of environmental variables with variance inflation factors >10, the numbers of variables per dataset used in the RDA analyses were reduced to an average of 10.9 ± 3.0 (range: 4–16) with a total of 61 variables. The final environmental RDA models included 2.6 ± 0.9 predictor variables per dataset and a total of 30 variables. Site elevation and stream width were usually important in explaining variation within metacommunities. However, some variables available in many datasets were never (e.g., SO_4_ and dead wood material) or seldom selected in the models (e.g., total phosphorus and conductivity; Appendix S5). On the other hand, multivariate models selected a few variables more often than would have been expected from their proportional availability (e.g., bank modification, clogging, and stream order). These variables, however, were available for few datasets (<4), making it difficult to determine their general importance in structuring the metacommunities.

## Discussion

Our findings showed that variation in the beta diversity of stream insects *across* the 95 metacommunities (i.e., spatial level 1) was not well explained by ecological predictors, such as insect group, spatial extent, latitude, and altitudinal range. In addition, environmental, and spatial variables were poor predictors of assemblage composition *within* most metacommunities (i.e., spatial level 2), although environmental variables tended to be better predictors than spatial ones. Because our findings were consistent across a large number of datasets from around the world, we believe that they represent worldwide patterns in stream insect metacommunities. Below, we will discuss our finding in the context of spatial extent, environmental heterogeneity, latitude, and predictability.

Beta diversity is expected to increase with increasing spatial extent for four reasons (Bini et al. 2014; Heino et al. 2015b). First, larger areas encompass higher environmental heterogeneity than small areas. Therefore, an increase in environmental heterogeneity is hypothesized to be positively related to the strength of species sorting processes, although evidence for such a relationship is scant (Landeiro et al. 2012; Grönroos et al. 2013). Second, the effect of dispersal limitation, promoting differences in species composition among sites, is expected to increase with spatial extent (Cottenie 2005; Heino 2011). Third, a positive relationship between beta diversity and spatial extent may arise from sampling different regional species pools (Heino et al. 2015a). Fourth, the relationship between beta diversity and spatial extent is also expected due to a negative relationship between pairwise similarity in assemblage composition and geographic distance (i.e., the distance decay of similarity; Nekola and White 1999). However, our findings did not support any of these mechanisms underlying variability in beta diversity, despite the relatively large variation in spatial extent among the metacommunities studied (measured as average distance to group geographical centroid: min = 0.39 km, max = 125.06 km). Similarly, altitudinal range, an important predictor of biodiversity patterns (Bini et al. 2004; Melo et al. 2009), was a poor predictor of beta diversity in our study. This was surprising because the drainage basins we studied ranged from lowland to montane areas (range of altitudinal range: min = 11 m a.s.l., max = 2136 m a.s.l.), potentially involving considerable variation in environmental factors that affect stream insect distributions between streams located at low and high altitudes (e.g., temperature, flow, and substratum; see Ward 1998; Jacobsen and Dangles 2012).

According to Rapoport's rule, species ranges tend to be larger at high latitudes (Stevens 1989), and this phenomenon may give rise to a latitudinal gradient in beta diversity (Soininen et al. 2007). However, latitude was a poor predictor of beta diversity *across* the metacommunities. It seems that patterns in alpha, beta, and gamma diversity of aquatic insects do not necessarily match those of mammals, birds, or vascular plants (Boyero et al. 2011; Heino 2011). While many terrestrial taxa show pronounced latitudinal gradients (Stevens 1989), gamma diversities of mayflies and stoneflies both peak at mid- to high latitudes, that of caddisflies show no significant relationship with latitude, and that of dragonflies show a latitudinal cline typical of those observed in most terrestrial taxa (Pearson and Boyero 2009). Furthermore, alpha diversities of mayflies, stoneflies, and caddisflies do not follow typical latitudinal clines (Vinson and Hawkins 2003). Although knowledge of global variation in beta diversity of stream insects is limited, information about their gamma and alpha diversities suggests that they are unlikely to exhibit clear latitudinal gradients in beta diversity (Pearson and Boyero 2009; Boyero et al. 2011). Moreover, even when alpha and gamma diversity show a latitudinal cline, such a cline may not necessarily be expected in beta diversity, because different diversity components may vary independent of each other spatially and temporally (Angeler and Drakare 2013).

A salient finding was that dataset characteristics were the main predictors of variation in beta diversity *across* the 95 metacommunities. Although it seems obvious that matrix fill should be negatively related to beta diversity, our results clearly demonstrate the importance of taking this data property into account when attempting to find correlates of beta diversity. This issue has rarely been considered and we suggest that taking dataset characteristics into account as covariables, before conjecturing about ecological explanations, should be standard practice in future comparative studies.

A combination of low matrix fill (i.e., numerous absences in the site-by-species matrix) and large numbers of rare species typically results in high beta diversity values (Podani and Schmera 2011). Rare species are a typical property of stream insect datasets (Malmqvist et al. 1999; Heino 2005). This may be a result of the high variability in stream hydrology. Such variability makes the distributions of individual rare species more difficult to model than the distributions of individual common species (Soininen et al. 2013) and, hence, variation in species composition may likewise be highly challenging to model at the assemblage level.

Streams are dynamic systems due to recurrent high or low flows (Resh et al. 1988; Townsend 1989; McGarvey 2014), and this variability in flow affects habitat conditions. Such disturbance may temporarily eliminate species from a site, leading to unexpected absences at sites that are otherwise environmentally suitable (Heino and Peckarsky 2014). If such disturbances are frequent, one is likely to find a large number of rare species in a metacommunity at a given point in time. Streams are also highly heterogeneous at various spatial scales (Leung and Dudgeon 2011; Heino et al. 2013), promoting ecological specialization and leading to a high number of rare species in stream insect metacommunities (Heino 2005; Allan and Castillo 2007). Thus, high frequencies of rare species in stream insect metacommunities may not only stem from high variability in stream hydrology but also be enhanced by high environmental heterogeneity. Although the relationships between flow variability and different response variables (e.g., abundance and diversity) have been extensively studied (see review in Poff and Zimmerman 2010), we are not aware of any studies that have examined the effects of flow variability on beta diversity. We believe that testing the relationship between beta diversity and flow variability would be a fruitful idea for future research.

Even in cases where predictor variables accounted for statistically significant amounts of variation in assemblage composition *within* metacommunities, the proportions of variation they explained were rather low. A low proportion of explained variation has been a typical finding in recent studies of stream and other metacommunities using modern analytical methods comparable to those used in our study (Beisner et al. 2006; Nabout et al. 2009; Landeiro et al. 2012; Alahuhta and Heino 2013; Göthe et al. 2013; Grönroos et al. 2013). The robustness of this finding across the world suggests that the structure of stream insect metacommunities shows low predictability. At the very least, the environmental variables that researchers typically measure in ecological studies may not always account for a large proportion of variation in the assemblage composition of stream insects. The low explanatory power of environmental predictors may stem from the possibility that single snapshot sampling of biological assemblages and environmental variables fails to reveal strong assemblage–environment relationships (Beisner et al. 2006; Erős et al. 2012). If assemblage–environment relationships generally vary in time (Heino and Mykrä 2008; Erős et al. 2012), then this may have major consequences for applied ecology.

Although our predictor variables were important in about half of the datasets and the proportion of variation explained in all cases was low (<50%), environmental variables tended to have a more important influence on stream insect metacommunity structure than spatial variables. This result agrees with previous studies restricted to a single or a few drainage basins (Landeiro et al. 2012; Al-Shami et al. 2013a; Göthe et al. 2013; Grönroos et al. 2013). Furthermore, among the many environmental variables included in our study, we found that some easily obtainable variables had particular explanatory power, including stream elevation and stream width. On the other hand, some frequently measured variables (Appendix S3 and S5) were never selected in the models and thus only seem relevant in very specific regional contexts.

Environmental assessment and biodiversity conservation are often based on indicator taxon groups (Caro 2010). In running waters, mayflies, stoneflies, and caddisflies have typically been considered as sensitive indicators of environmental degradation (Rosenberg and Resh 1993), whereas dragonflies have been proposed as a candidate indicator group of overall biodiversity, especially in the tropics (Simaika and Samways 2011). To be efficient in either task, different taxa should be indicators of changes in environmental conditions and variation in their biodiversity should show congruence with other groups (Caro 2010). Although we did not aim to directly examine cross-taxon congruence in biodiversity patterns, it is worth noting that patterns exhibited by single taxonomic groups of the five studied did not necessarily match those of other taxa, which may limit their use as biodiversity indicators at broad spatial scales.

There are three limitations in our current study. First, although our insect data were resolved to the lowest possible level of identification in each drainage basin, taxonomic resolution varied to some extent among the metacommunities sampled. We controlled for this by a variable describing the percentage of taxa identified to the species level in the comparative analysis, but this variable did not show a significant relationship with variation in beta diversity *across* the metacommunities. Second, we cannot rule out the possibility that unmeasured ecological variables are the main drivers of variation in species composition *within* each metacommunity. Potentially important variables include fish predation, primary production, and proxies of stream-bed disturbance, which have been shown to be important drivers in single case studies (e.g., Townsend et al. 2000). Obtaining such site-level variables that are strictly comparable across datasets, however, was impossible given our broad-scale approach. Third, we were limited by data availability to considering only five taxonomic groups, all of which shared some characteristics (e.g., all had flying adult stages). We cannot predict how patterns of beta diversity may play out for groups that are restricted to in-stream dispersal (e.g., fish, mollusks, crayfish, and shrimps).

An important consideration related to the second and third limitations of our study is that spatial variables derived from watercourse and overland distances might have different predictive power in explaining variation in species composition within each drainage basin (Jacobson and Peres-Neto 2010; Altermatt 2013; Altermatt et al. 2013). Stream insects disperse from one stream to another by following watercourses or crossing land between headwater streams (Malmqvist 2002; Lancaster and Downes 2013). Thus, it is not clear which dispersal route is more strongly associated with variation in insect species composition among streams. Previous studies have suggested that there is little difference between the predictive power of spatial variables derived from watercourse or overland distances if one uses robust spatial analysis methods, such as Moran eigenvector maps (Landeiro et al. 2012; Göthe et al. 2013; Grönroos et al. 2013). We believe that the Moran's eigenvector map approach used would have modeled spatial structuring of stream insect assemblage composition adequately if, indeed, such structuring existed. Furthermore, using multiple potentially efficient spatial variables did not lead to additional predictive power in our analyses within drainage basins.

In general, the low explanatory power we found in analyses of beta diversity is in line with the comprehensive study of Low-Décarie et al. (2014). They evaluated 18 000 ecological articles and found a temporal decline in the coefficient of determination in research studies. We favor one of the three hypothesis proposed by Low-Décarie et al. (2014) to explain this trend: “The low hanging fruit hypothesis proposes that simple discoveries are made early in the development of a discipline and what remains to be explained, at the margins, is increasingly complicated and difficult to reach. In ecology, there appears to be a trend away from single species studies toward more complex community studies, as well as less emphasis on topics that are more observational and arguably less dependent on statistics, such as behavior and physiology, with concurrent increases in statistically complex topics such a biodiversity.” Alternatively, the low explanatory power in our analyses may indicate that we should increase our effort in measuring more difficult, albeit potentially more relevant, predictor variables during field surveys.

Our main finding is that variation in the beta diversity of stream insects is difficult to predict *across* metacommunities and *within* each metacommunity. This finding may be related to frequent disturbances in stream systems, resulting in large numbers of rare species in local communities. The weak relationships between beta diversity and latitude, altitudinal range and spatial extent also suggest that stream insects do not follow the geographical patterns observed in alpha, beta, and gamma diversity of various terrestrial taxa. Our comparative analysis of stream insect metacommunities thus (i) reveals a deviance from the general distribution of biodiversity across the world and (ii) calls for reconsideration of the predictability of the responses of stream insect assemblages to ecological gradients.

## Supporting Information

**Appendix S1.** A schematic figure showing the spatial level 1 of our analyses: across multiple metacommunities. **Appendix S2.** A schematic figure showing the spatial level 2 of our analyses: within each metacommunity. **Appendix S3.** Environmental variables available and the frequency of datasets in which they appeared. **Appendix S4.** Relationship between Sørensen beta diversity and latitude for the five insect taxa. **Appendix S5.** Frequency of environmental variables in datasets and frequency at which they were selected in RDA or pRDA models.
