# Supplementary material for: A comparative analysis reveals weak relationships between ecological factors and beta diversity of stream insect metacommunities at two spatial levels
Source: Ecol Evol. 2015 Feb 23;5(6):1235–48. doi: 10.1002/ece3.1439 (PMC4377267; doi:10.1002/ece3.1439)
Supplement: Supplementary file 1 [file ece30005-1235-sd1.docx]

*Ecology and Evolution*

**Supporting information**

**A comparative analysis reveals weak relationships between ecological factors and beta diversity of stream insect metacommunities at two spatial levels**

Jani Heino, Adriano S. Melo, Luis Mauricio Bini *et al*.

**Appendix S1.** A schematic figure showing spatial level 1 of our analyses: across multiple metacommunities (MC). These analyses used a dissimilarity-based approach, where averages of pairwise dissimilarities were (1) calculated for each metacommunity (n = 95 metacommunities) and (2) the values of beta diversity were used in a comparative analysis with insect group, spatial extent, altitude range, latitude and dataset properties as predictors.

**Appendix S2.** A schematic figure showing spatial level 2 of our analyses: within each metacommunity (MC). These analyses used the raw data approach, where we analysed data on insect assemblages (Bio), environmental variables (Env) and spatial (Spa) variables taken from each stream site within a drainage basin (n = 61 metacommunities). Variation in insect assemblage structure was partitioned into four fractions: [a] = pure environment, [b] = shared environmental and spatial effects, [c] = pure space and [d] = unexplained.

**Appendix S3.** Environmental variables available and the frequency of datasets in which they appeared. A total of 61 datasets from 20 basins were available. Each of the 20 basins included 1 to 5 datasets representing distinct insect groups (Ephemeroptera, Plecoptera, Trichoptera, Chironomidae and Odonata). Some related variables are listed separately as they were measured in distinct ways in different studies: these related (or even similar) variables did not affect the study, because they were used separately for *within* metacommunity analyses. Freq = frequency.

| Variable | Freq |  | Variable | Freq |  | Variable | Freq |
| --- | --- | --- | --- | --- | --- | --- | --- |
| Conductivity | 54 |  | K | 13 |  | Silica | 4 |
| Total P | 41 |  | OH^-^ | 13 |  | Stream order | 4 |
| Depth | 40 |  | Roots | 13 |  | Fluoride | 3 |
| Width | 39 |  | Undercut | 13 |  | Nitrate.Nitrite | 3 |
| Shading | 39 |  | Cobble | 12 |  | Orthophosphate | 3 |
| Elevation | 38 |  | Total dissolv. solids | 10 |  | Sodium | 3 |
| pH | 37 |  | Total suspend. solids | 9 |  | Total Fe | 3 |
| Water temperature | 35 |  | Color | 8 |  | Absorbance | 3 |
| Total N | 33 |  | Cl | 7 |  | Acid-neutraliz. capacity | 3 |
| Boulder | 30 |  | Fast flow percent | 6 |  | TOC | 3 |
| Macrophyte cover | 28 |  | Chlorophyll a | 6 |  | Algae | 3 |
| Alkalinity | 26 |  | Substrate > 16mm | 6 |  | Clogging | 3 |
| Velocity | 23 |  | Periphyton | 6 |  | Embankment modif. | 3 |
| Turbidity | 22 |  | Detritus | 5 |  | FeS | 3 |
| SO4 | 21 |  | Pheophytin | 5 |  | Foam | 3 |
| Dead wood | 20 |  | Clear water | 4 |  | Moss | 3 |
| Discharge | 17 |  | CPOM | 4 |  | Mud | 3 |
| Substrate | 17 |  | Slope | 4 |  | River bed modified | 3 |
| Dissolved oxygen | 16 |  | Ca | 4 |  | NO_3_ | 1 |
| Mg | 16 |  | HCO3 | 4 |  | Oxygen | 1 |
| Filamentous algae | 13 |  | Kjeldahl nitrogen | 4 |  | SRP | 1 |
| Artificial structures | 13 |  | KOI Cr | 4 |  |  |  |
| H+ | 13 |  | Loss on ignition | 4 |  |  |  |

**
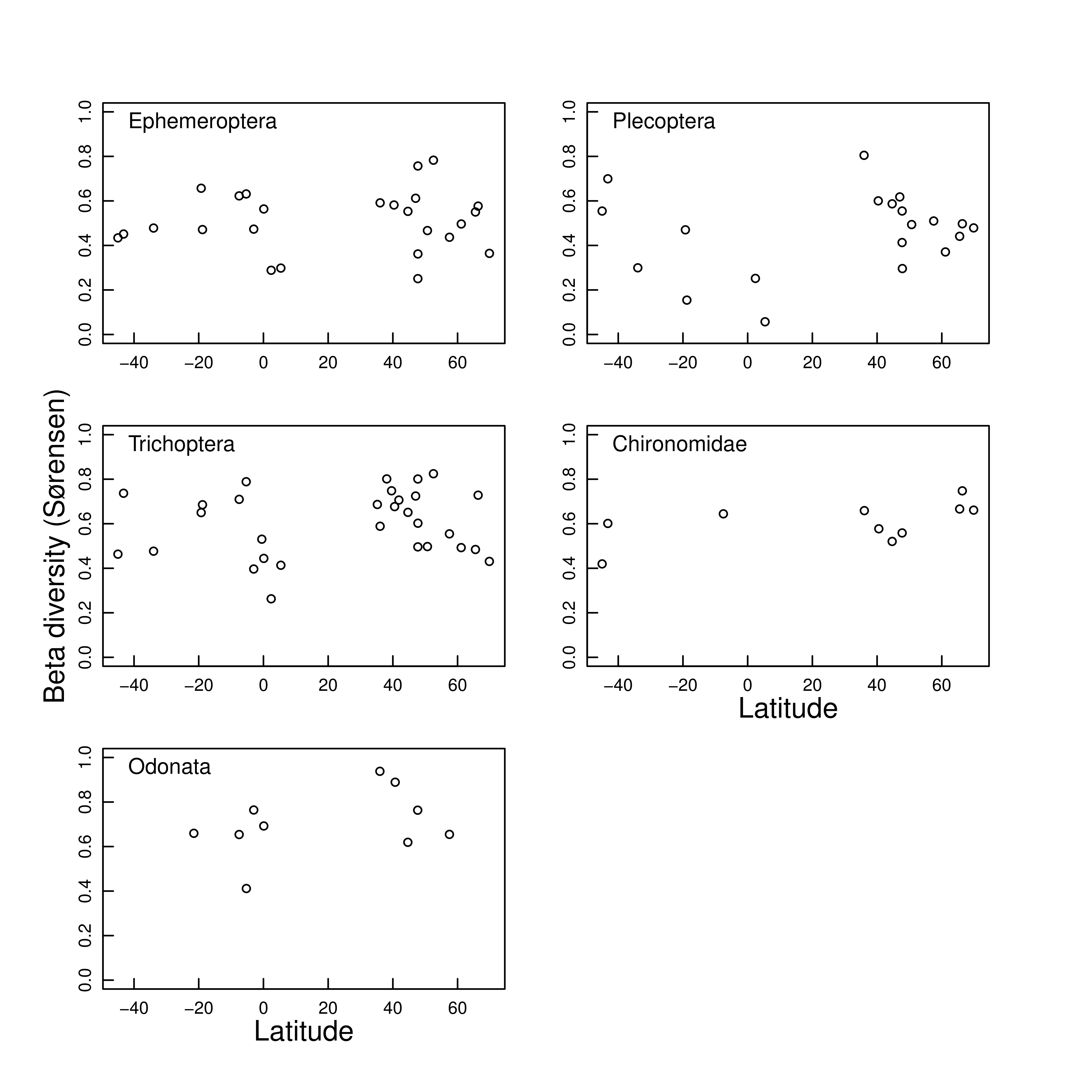
**

**Appendix S4.** Relationships between Sørensen beta diversity and latitude for the five insect taxa.

**
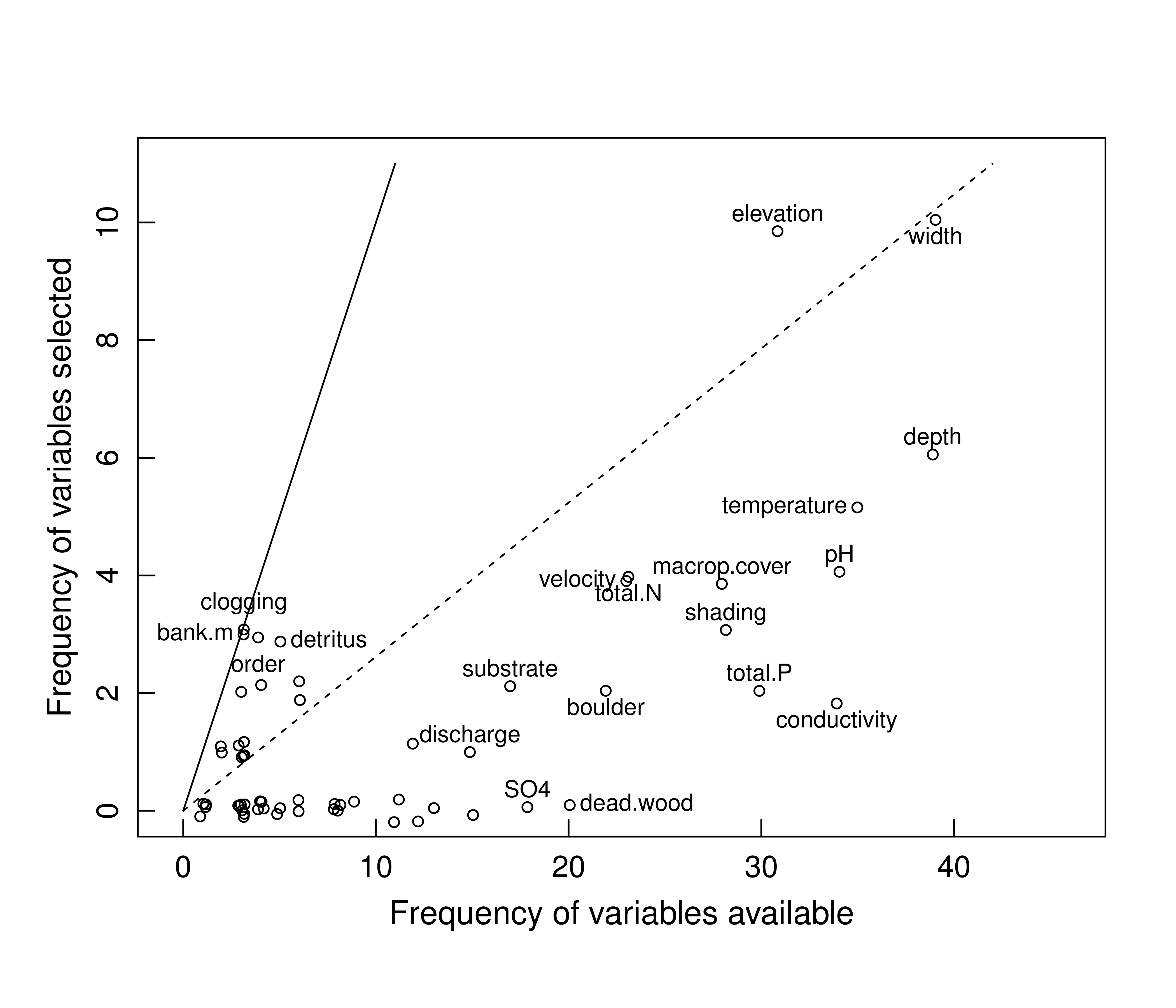
**

**Appendix S5**. Frequency of environmental variables in datasets and frequency at which they were selected in RDA or pRDA models. Variables on the dashed line were selected in the same proportion as they were available. Variables on the solid line were selected in all instances they were available (order = stream order; bank.m = stream bank modification). Some symbols have been shifted slightly to avoid overlap.
